# Impact of Euploid Embryo Transfer and Maternal Age on Implantation Outcomes in In Vitro Fertilization-Embryo Transfer (IVF-ET): A Systematic Review

**DOI:** 10.7759/cureus.109784

**Published:** 2026-05-27

**Authors:** Rania E Belal, Suhila Badwey Majzoub Karamalla, Sara Kheiry, Rashad El-Maddah Mohamed Mostafa Gamea, Saeed Saad Saeed Alatam, Ehab A Elagab

**Affiliations:** 1 Obstetrics and Gynecology, Mouwasat Hospital, Jubail, SAU; 2 Obstetrics and Gynecology, King Faisal Specialist Hospital and Research Centre, Madinah, SAU; 3 Obstetrics and Gynecology, University of Khartoum, Khartoum, SDN; 4 Obstetrics and Gynecology, Noonu Atoll Hospital, Manadhoo Island, MDV; 5 Emergency Medicine, Najran Armed Forces Hospital, Ministry of Defense Health Services, Najran, SAU; 6 Pathology, College of Medicine, Najran University, Najran, SAU

**Keywords:** euploid embryo transfer, implantation rate, ivf-et, live birth rate, maternal age, pgt-a, recurrent pregnancy loss

## Abstract

Maternal age is a well-established determinant of IVF success, primarily due to increased embryonic aneuploidy. Preimplantation genetic testing for aneuploidy (PGT-A) enables the selection of euploid embryos, potentially improving implantation and live birth rates. However, whether euploid embryo transfer can fully overcome the negative impact of advancing maternal age remains uncertain. This systematic review aims to evaluate the impact of euploid embryo transfer and maternal age on implantation and pregnancy outcomes in IVF-ET cycles.

A comprehensive literature search was conducted across PubMed, Scopus, Web of Science, and Embase for studies published between 2021 and 2025. Eligible studies were retrospective cohort studies reporting implantation, clinical pregnancy, or live birth outcomes following euploid embryo transfer across different maternal age groups. The PICOS framework guided eligibility criteria. Risk of bias was assessed using the ROBINS‑I tool. A narrative synthesis was performed due to heterogeneity in study designs, age cut-offs, and outcome definitions.

Eight retrospective cohort studies encompassing 7,537 patients were included. Three studies reported a significant decline in live birth rates with advancing maternal age, despite euploid transfer, particularly in women ≥38 years, with miscarriage rates as high as 22.6% in the oldest group. Two studies found no independent age effect after adjusting for embryo morphology and endometrial preparation. Euploid transfer demonstrated marked benefit in recurrent pregnancy loss patients (live birth rate 80% vs. 0% in controls) but no benefit in recurrent implantation failure. Blastocyst trophectoderm grade was a stronger predictor of live birth than maternal age in two studies. Oocyte origin (autologous vs. donor) did not affect outcomes in advanced maternal age patients. Six studies had low overall risk of bias, while two showed moderate-to-serious risk.

Euploid embryo transfer significantly improves implantation and reduces miscarriage rates compared to untested embryos; however, maternal age continues to influence live birth outcomes in women aged 38 years and older. The benefit of PGT-A varies by clinical indication: strongly supported for recurrent pregnancy loss, equivocal for advanced maternal age, and not supported for recurrent implantation failure. Blastocyst morphology remains prognostically important even after ploidy selection. Prospective studies with standardized age cut-offs and cumulative live birth reporting are needed to refine patient selection for PGT-A.

## Introduction and background

Infertility has emerged as a significant and increasing global public health concern, affecting a considerable proportion of reproductive-aged couples worldwide. In response, in vitro fertilization (IVF) combined with embryo transfer (ET) has become one of the central and most effective assisted reproductive technologies used to manage infertility and achieve pregnancy [1]. However, despite substantial progress in laboratory culture systems, stimulation protocols, and embryo transfer techniques, implantation failure continues to represent a major bottleneck limiting overall IVF success rates and remains a key clinical challenge in reproductive medicine [2]. Among the multifactorial determinants of implantation success, embryo chromosomal status and maternal age are widely recognized as two of the most influential and biologically interconnected factors.

Embryo aneuploidy, defined as an abnormal number of chromosomes, is one of the primary causes of failed implantation, early pregnancy loss, and reduced live birth rates in IVF cycles [3]. To address this limitation, preimplantation genetic testing for aneuploidy (PGT-A) has been introduced into clinical practice, allowing the identification and selection of euploid embryos with a normal chromosomal complement prior to transfer [4]. This advancement has led to a shift toward euploid embryo transfer strategies, which are intended to improve implantation potential, reduce miscarriage risk, and optimize reproductive outcomes, particularly in patients with recurrent implantation failure, advanced maternal age, or diminished ovarian reserve [5]. Despite its increasing clinical adoption, the true extent to which euploid selection alone can optimize implantation outcomes across different patient populations remains an area of ongoing investigation.

Maternal age is another well-established determinant of reproductive success in assisted reproduction, with a progressive decline in fertility observed particularly after the age of 35 years [6]. This decline is strongly associated with increased oocyte aneuploidy rates, reduced embryo developmental competence, and overall diminished reproductive potential [7]. However, emerging evidence suggests that the impact of maternal age may extend beyond oocyte quality alone, potentially influencing endometrial receptivity, uterine vascular function, and the molecular environment required for successful implantation. Consequently, even in cycles where euploid embryos are transferred, maternal age may continue to exert an independent effect on implantation and pregnancy outcomes [8].

Despite advances in embryo selection strategies, the relative contribution and interaction between euploid embryo transfer and maternal age in determining implantation success remain controversial. Some studies propose that the transfer of chromosomally normal embryos may largely overcome the negative impact of advanced maternal age, resulting in comparable implantation and pregnancy outcomes across age groups [5]. In contrast, other reports demonstrate persistent age-related differences in implantation rates and reproductive outcomes even after euploid embryo transfer, suggesting that maternal age may have additional biological effects beyond embryo chromosomal status [9]. These inconsistencies highlight a critical gap in the literature and underscore the need for a comprehensive synthesis of existing evidence.

Therefore, this systematic review aims to evaluate and synthesize current evidence on the impact of euploid embryo transfer and maternal age on implantation outcomes in IVF-ET cycles. By critically analyzing implantation rates, clinical pregnancy rates, and live birth outcomes across different maternal age groups, this review seeks to clarify whether euploid embryo selection can fully mitigate age-associated reproductive decline or whether maternal age continues to exert an independent influence on implantation success.

## Review

Methods

Study Design

This systematic review was conducted in accordance with the Preferred Reporting Items for Systematic Reviews and Meta-Analyses (PRISMA) guidelines [10] to ensure transparency, reproducibility, and methodological rigor in the identification, selection, and synthesis of evidence. The review protocol was developed a priori, defining the research question, eligibility criteria, and analytical approach to minimize bias in study selection and data interpretation.

Eligibility Criteria (PICOS Framework)

The eligibility criteria were defined using the PICOS framework [11]. The population (P) included women undergoing IVF or intracytoplasmic sperm injection (ICSI) cycles with embryo transfer. The intervention (I) was euploid embryo transfer following PGT-A. The comparator (C) included different maternal age groups, typically stratified as younger versus advanced maternal age, or age-based subgroups as defined by individual studies. The outcomes (O) of interest included implantation rate, clinical pregnancy rate, and live birth rate. The study design (S) included observational studies, cohort studies, retrospective analyses, and randomized controlled trials that reported relevant reproductive outcomes in relation to euploid embryo transfer and maternal age. Only studies published in English between 2021 and 2025 were included to ensure that the synthesis reflects the most recent advances in assisted reproductive technologies and genetic screening methods, while older studies were excluded due to potential methodological and technological differences in PGT-A platforms and embryo culture systems.

Information Sources

A comprehensive literature search was conducted across four major electronic databases: PubMed/MEDLINE, Scopus, Web of Science, and Embase. These databases were selected due to their extensive coverage of biomedical, reproductive medicine, and clinical research literature, ensuring a broad and systematic capture of relevant studies. The search was supplemented by manual screening of reference lists from included studies to identify any additional eligible articles that might have been missed during the database search.

Search Strategy

A structured search strategy was developed using a combination of controlled vocabulary (e.g., MeSH terms) and free-text keywords related to “euploid embryo transfer,” “preimplantation genetic testing,” “PGT-A,” “maternal age,” “IVF,” “implantation rate,” and “clinical pregnancy.” Boolean operators (AND/OR) were used to combine search terms appropriately across databases. The search strategy was tailored for each database to maximize sensitivity and specificity. The final search was restricted to studies published from 2021 to 2025.

Study Selection

All retrieved records were imported into EndNote X21 reference management software, where duplicate records were systematically identified and removed. Following deduplication, titles and abstracts were independently screened against the eligibility criteria. Full-text articles of potentially relevant studies were then retrieved and assessed for final inclusion. Any disagreements during the screening process were resolved through discussion and consensus to ensure consistency in study selection.

Data Collection Process and Data Items

Data from the included studies were systematically extracted using a standardized data extraction form. The extracted variables included study characteristics (author, year, country, and study design), participant characteristics (sample size and maternal age distribution), intervention details (IVF/ICSI protocol and use of PGT-A), embryo characteristics (euploid embryo transfer), and outcome measures (implantation rate, clinical pregnancy rate, and live birth rate). Where necessary, study authors’ definitions of outcomes were retained to maintain methodological consistency with original reports.

Risk of Bias Assessment

The methodological quality and risk of bias of the included non-randomized studies were assessed using the Risk Of Bias In Non-randomized Studies of Interventions (ROBINS-I) tool [12]. This instrument evaluates bias across multiple domains, including confounding, participant selection, classification of interventions, deviations from intended interventions, missing data, outcome measurement, and selective reporting. Each study was categorized as having low, moderate, serious, or critical risk of bias based on domain-level judgments.

Synthesis of Results

A narrative synthesis approach was used to summarize and interpret the findings across included studies. Due to significant heterogeneity in study designs, maternal age categorizations, PGT-A technologies, embryo transfer protocols (fresh versus frozen cycles), and outcome definitions, a quantitative meta-analysis was not performed. Additionally, variability in reporting implantation and pregnancy outcomes, along with differences in statistical adjustments for confounders across studies, limited the comparability of effect sizes. Therefore, a structured qualitative synthesis was deemed more appropriate to avoid misleading pooled estimates and to preserve the clinical and methodological context of individual studies.

Results

Study Selection Process

**Figure 1:**
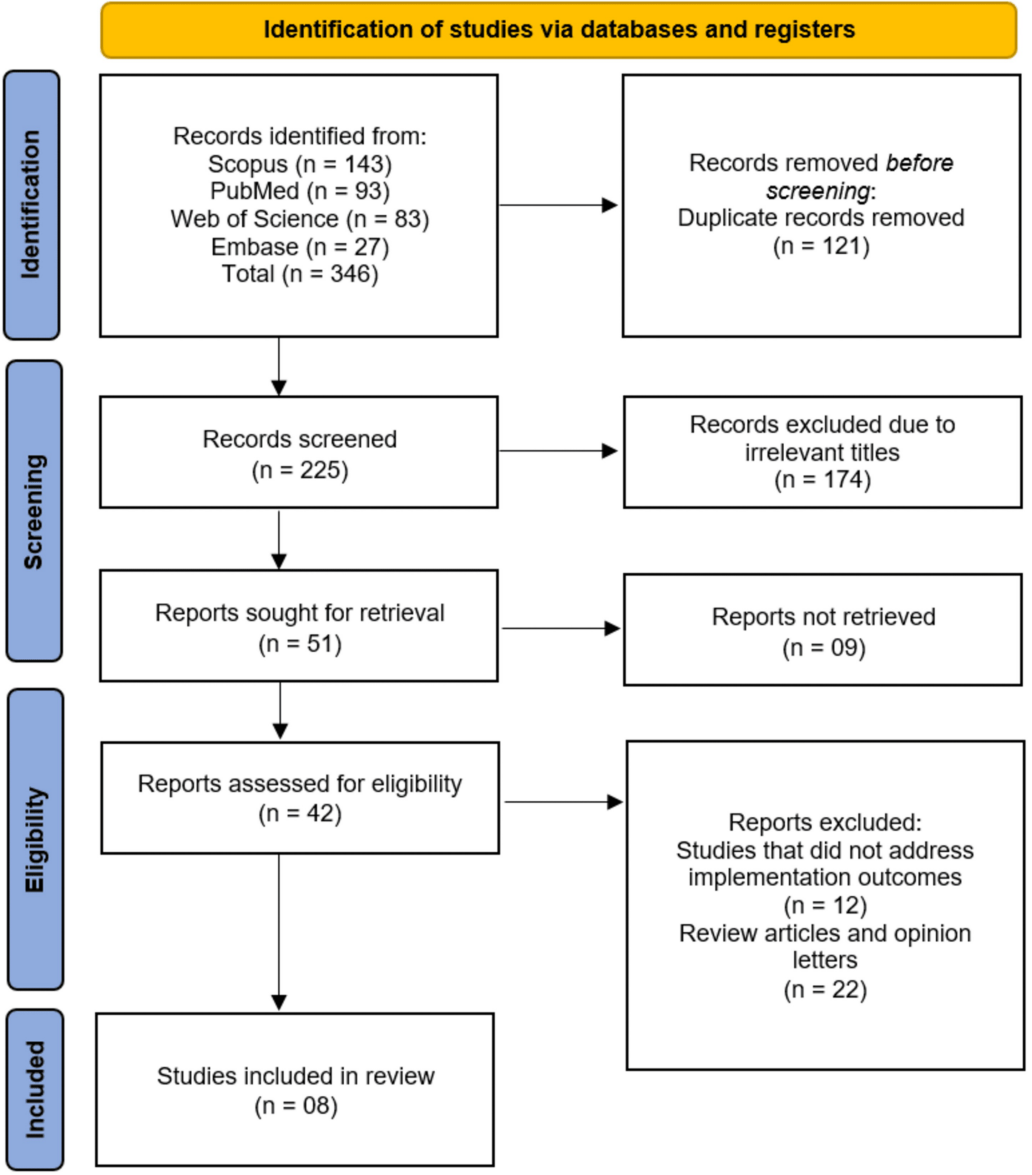
PRISMA Flowchart of Studies Selection Process

The study selection process followed the Preferred Reporting Items for Systematic Reviews and Meta-Analyses (PRISMA) guidelines, as detailed in the PRISMA flowchart. A total of 346 records were identified from four electronic databases: Scopus (n = 143), PubMed (n = 93), Web of Science (n = 83), and Embase (n = 27). After removing 121 duplicate records, 225 records remained for title screening. Of these, 174 records were excluded due to irrelevant titles, leaving 51 reports sought for retrieval. Nine reports could not be retrieved, resulting in 42 reports assessed for full-text eligibility. Following full-text review, 12 studies were excluded because they did not address implementation outcomes, and 22 reports were excluded as they were review articles or opinion letters. Consequently, a total of eight studies [13-20] met the inclusion criteria and were included in this systematic review (Figure 1).

Characteristics of the Included Studies

A total of eight retrospective cohort studies, published between 2021 and 2025, met the inclusion criteria for this systematic review. The characteristics of these studies are summarized in Table 1. The studies were conducted across diverse geographical settings, including China [13,14,19], Spain and Italy [15], the UAE [16], Japan [17], Greece [18], and Spain alone [20]. Sample sizes varied considerably, ranging from 273 patients [19] to 2,588 patients [17]. All studies employed single euploid embryo transfer (SET) except Pantou et al. [18], who predominantly used double embryo transfer (DET) with one to three embryos. Maternal age cut-offs or ranges differed across studies, with advanced maternal age (AMA) typically defined as ≥35 or ≥38 years. Regarding IVF protocols, all studies utilized FET except Tan et al. [20] (fresh vs. FET) and Pantou et al. [18] (fresh cycles). PGT-A methods included next-generation sequencing (NGS) [13-16], array comparative genomic hybridization (aCGH) [17,18], and noninvasive chromosome screening (NICS) [19]. Endometrial preparation protocols varied, including natural cycles, hormone replacement therapy (HRT), and individualized protocols, which may have contributed to between-study heterogeneity. Blastocyst-stage transfer (day 5-6) was standard across all studies, with Chen et al. [14] also including day seven blastocysts.

**Table 1:** Characteristics of Included Studies aCGH: array comparative genomic hybridization; AMA: advanced maternal age; CC: citrate cocktail; COS: controlled ovarian stimulation; CP: clinical pregnancy; CPR: clinical pregnancy rate; DET: double embryo transfer; E2: estradiol; EPL: early pregnancy loss; ET: embryo transfer; FET: frozen embryo transfer; GA: gestational age; GnRH: gonadotropin-releasing hormone; GnRH-a: GnRH agonist; HRT: hormone replacement therapy; ICSI: intracytoplasmic sperm injection; IR: implantation rate; IVF: in vitro fertilization; LBR: live birth rate; MC: miscarriage; MR: miscarriage rate; NGS: next-generation sequencing; NICS: noninvasive chromosome screening; NR: not reported; OPR: ongoing pregnancy rate; PGT-A: preimplantation genetic testing for aneuploidy; PGT-M: preimplantation genetic testing for monogenic disorders; PGT-SR: preimplantation genetic testing for chromosomal structural rearrangements; PSM: propensity score matching; RIF, recurrent implantation failure; RM: recurrent miscarriage; RPL: recurrent pregnancy loss; SET: single embryo transfer; TE: trophectoderm.

First Author (Year)	Country	Study Design	Sample Size (Patients/Cycles)	Maternal Age (Mean ± SD / Range)	IVF Protocol (Fresh/FET, PGT-A Method)	Embryo Type	Stage of Transfer (Cleavage/Blastocyst)	Endometrial Preparation	Outcomes Reported
Jiang et al., [13] (2025)	China	Retrospective cohort	1037 cycles	<35 (n=796), 35–37 (n=126), ≥38 (n=115)	FET; PGT-A via NGS (±PGT-M/SR); ICSI	SET	Blastocyst (D5–D6)	Individualized (GnRH-a/antagonist protocols)	Clinical pregnancy, EPL, miscarriage, live birth
Chen et al., [14] (2025)	China	Retrospective cohort	941 / 538 FET	34.28 ± 5.08 y	ICSI; FET; NGS (24-chr PGT-A)	SET	Blastocyst (D5–7)	Natural + HRT (±GnRH agonist)	Euploidy rate; LBR; morphology effect
Cozzolino et al., [15] (2024)	Spain & Italy	Retrospective multicenter cohort	556 cycles (278 autologous, 278 donor)	41.74 ± 1.91 / 41.98 ± 3.92 (39–46 yrs)	FET; PGT-A (NGS)	SET	Blastocyst (D5–D6)	HRT or natural FET cycle (E2 + progesterone ± GnRH agonist)	Implantation, biochemical pregnancy, ectopic pregnancy, miscarriage, LBR, GA, birth weight
Lawrenz et al., [16] (2024)	UAE	Retrospective cohort	1464 / 1923	Median 34 (29–38)	FET; PGT-A by NGS	SET (euploid)	Blastocyst (D5–6)	Natural cycle, HRT	OPR, miscarriage, LBR
Kato et al., [17] (2023)	Japan	Retrospective cohort (PSM)	2588 pts (25 PGT-A vs controls matched)	35–42 yrs	FET; aCGH PGT-A	SET	Blastocyst (D5–6)	NR	Implantation, CP, MC, LBR, neonatal, child development
Pantou et al., [18] (2022)	Greece	Retrospective cohort (PGT-A vs control)	455 couples; 744 ET cycles	AMA: 41.3 (37–49); RM: 35.9 (31–45); RIF: 34.7 (29–39)	Fresh IVF; ICSI; aCGH-based PGT-A	Mostly DET (1–3 embryos)	Blastocyst (D5–6)	COS (GnRH agonist/antagonist/CC)	Implantation, LBR, miscarriage, biochemical loss, ectopic, aneuploidy
Xi et al., [19] (2022)	China	Retrospective cohort	273 / 407 FET	32–33 yrs (≈32.1–33.4 ± 3.8–4.7)	FET; NICS (NGS, spent media) vs morphology	SET	Blastocyst (D5–D6)	Vitrified-thawed FET cycle	CPR, MR, OPR, LBR, ectopic
Tan et al., [20] (2021)	Spain	Retrospective cohort	728 (339/389 cycles)	<43 yrs	Fresh vs FET; GnRH antagonist; PGT-A (TE biopsy, day 5–6)	SET (some DET)	Blastocyst (D5–6)	Fresh: progesterone post-retrieval; FET: estradiol + progesterone (HRT)	IR, CPR, MR

Effect of Maternal Age on Live Birth and Pregnancy Outcomes After Euploid Embryo Transfer

The association between maternal age and reproductive outcomes following euploid embryo transfer was a primary focus of five studies, with detailed outcome data presented in Table 2. Jiang et al. [13] reported a significant decline in live birth rate (LBR) with advancing maternal age: LBRs were 54.5% in patients <35 years, 54.0% in those 35-37 years, but only 41.7% in women ≥38 years after a single euploid FET. The adjusted odds ratio (aOR) for LBR in women ≥38 years was 2.19-2.24 (p<0.05), and miscarriage rates were highest in the oldest group (22.6% vs. 13.0% in younger groups). In contrast, Lawrenz et al. [16] found no significant effect of female age on ongoing pregnancy rate (OPR) following single euploid FET, with OPRs ranging from 47.4% to 52.8% across age groups (p=0.534-0.640), suggesting that embryo quality and endometrial preparation may be more influential than maternal age alone. Chen et al. [14] similarly reported no significant association between maternal age and LBR (p=0.5936), noting that blastocyst trophectoderm (TE) grade was more predictive of live birth. Cozzolino et al. [15] compared autologous versus donor oocyte cycles in women with AMA and found no significant differences in implantation (RR 0.92, 95% CI 0.64-1.32), LBR (RR 1.03, 95% CI 0.84-1.25), or miscarriage rates (RR 0.99, 95% CI 0.85-1.14) following single euploid FET, implying that oocyte origin does not modify the age-related effect on euploid embryo outcomes. Pantou et al. [18] observed that despite euploid embryo selection, AMA patients had the lowest live birth efficiency (35.3%) compared to those with recurrent miscarriage (RM, 50.0%) or recurrent implantation failure (RIF, 47.8%), highlighting that AMA remains a negative prognostic factor even with PGT-A.

**Table 2:** Implantation and Pregnancy Outcomes by Euploid Embryo Transfer and Maternal Age aOR: adjusted odds ratio; AMA: advanced maternal age; BMI: body mass index; CI: confidence interval; Comp: comparable (outcome not significantly different from control/comparator group); CPR: clinical pregnancy rate; DET: double embryo transfer; ET: embryo transfer; FET: frozen embryo transfer; Impl: implantation; LBR: live birth rate; MC: miscarriage; MR: miscarriage rate; N/A: not applicable; NICS: noninvasive chromosome screening; NR: not reported; NS: not significant; OPR: ongoing pregnancy rate; OR: odds ratio; PGT-A: preimplantation genetic testing for aneuploidy; RIF: recurrent implantation failure; RM: recurrent miscarriage; RPL: recurrent pregnancy loss; RR: risk ratio; SET: single embryo transfer; sig.: significant; TE: trophectoderm; ↑: increase/increased; ↓: decrease/decreased.

First Author (Year)	No. of Euploid Embryos Transferred	Implantation Rate (%)	Clinical Pregnancy Rate (%)	Live Birth Rate (%)	Miscarriage Rate (%)	Effect Size (OR/RR, 95% CI)	p-value	Key Findings
Jiang et al., [13] (2025)	796 / 126 / 115	NR	62.7 / 61.9 / 53.9	54.5 / 54.0 / 41.7	13.0 / 12.8 / 22.6	LBR: aOR 2.19–2.24 (ref ≥38); MR: aOR 0.32–0.37	LBR <0.05 (others NS)	Older age (≥38) → ↓ live birth & ↑ miscarriage despite euploid transfer
Chen et al., [14] (2025)	538 FET cycles (single euploid)	NR	NR	NR	NR	Age: NS (vs LBR)	0.5936	Age not linked to LBR; embryo TE grade more predictive
Cozzolino et al., [15] (2024)	278 / 278	57.2 / 57.9	NR	42.5 / 41.0	14.4 / 16.2	RR: Impl 0.92 (0.64–1.32); LBR 1.03 (0.84–1.25); Mis 0.99 (0.85–1.14)	.93 / .79 / .63	No sig. diff. in implantation, LBR, or miscarriage between autologous vs donor euploid FET; outcomes similar in AMA
Lawrenz et al., [16] (2024)	1 (single euploid FET per cycle)	51.8 / 47.4 / 52.3 / 52.8 (OPR*)	NR	NR	NR	0.93–1.12 (ref ≤35)	0.534–0.640	No age effect on OPR; embryo quality, BMI, protocol more important
Kato et al., [17] (2023)	RIF: 10 ET; RPL: 5 ET	NR	RIF: Comp; RPL: Comp	RIF: Comp; RPL: 80 vs 0	RIF: Comp; RPL: 20 vs 100	NR	RIF: NS; RPL: 0.005 / 0.0098	Euploid ET no benefit in RIF; strong benefit in RPL (↑LBR, ↓MC)
Pantou et al., [18] (2022)	AMA: 51 ET; RM: 18 ET; RIF: 23 ET	AMA: 52.9; RM: 61.1; RIF: 69.6	AMA: 52.9; RM: 61.1; RIF: 69.6	AMA: 35.3; RM: 50.0; RIF: 47.8	RM: 18.2; RIF: 31.3; AMA: NR	NR	NR	Euploid transfer improved outcomes across all groups; highest implantation in RIF, lowest live birth efficiency in AMA despite euploid selection.
Xi et al., [19] (2022)	177 SET cycles	NR	RPL: 49.6%; RIF: 46.9%	RPL: 38.9%; RIF: 29.7%	RPL: 17.9%; RIF: 23.3%	MR OR 0.39 (0.16–0.95); LBR OR 2.53 (1.28–5.02); CPR OR 2.82 (1.20–6.66)	0.038; 0.008; 0.018	NICS ↑ outcomes; ↓ miscarriage; no age data
Tan et al., [20] (2021)	N/A (no PGT-A euploid transfer reported)	55.8/46.8/42.3 (fresh) vs 54.9/48.2/43.7 (deferred)	NR (similar trends)	NR	NR	NR	0.45 / 0.45 / 0.27	Implantation ↓ with age; no fresh vs deferred difference; no euploid-specific data

Implantation and Miscarriage Rates in High-Risk Populations

Several studies specifically examined implantation and miscarriage outcomes in patients with recurrent pregnancy loss (RPL) or recurrent implantation failure (RIF). Kato et al. [17] demonstrated a marked benefit of PGT-A in RPL patients, with an LBR of 80% in the PGT-A group versus 0% in controls and a miscarriage rate of 20% versus 100% (p=0.005). However, no significant benefit was observed for RIF patients, suggesting that the utility of PGT-A varies by clinical indication. Xi et al. [19] evaluated NICS in RPL and RIF patients undergoing SET and reported significantly improved outcomes, including a lower miscarriage rate (OR 0.39, 95% CI 0.16-0.95; p=0.038) and higher LBR (OR 2.53, 95% CI 1.28-5.02; p=0.008) compared to morphology-only selection. Pantou et al. [18] reported the highest implantation rate in RIF patients (69.6%) and the lowest in AMA patients (52.9%) despite euploid transfer, reinforcing that implantation potential depends on patient diagnosis beyond embryo ploidy. Tan et al. [20] found that implantation rates declined with advancing age in both fresh (55.8% to 42.3%) and deferred (54.9% to 43.7%) transfer cycles, although no significant difference was observed between fresh and deferred strategies (p>0.05 for all comparisons); however, this study did not specifically report euploid-confirmed transfers.

Other Predictors of Outcomes and Confounding Factors

Beyond maternal age, other factors influenced outcomes following euploid embryo transfer. Chen et al. [14] found that blastocyst morphology and developmental rate significantly affected euploidy and LBR, with TE grade being a stronger predictor than maternal age. Lawrenz et al. [16] identified body mass index (BMI) and endometrial preparation protocol as significant confounders for ongoing pregnancy. Cozzolino et al. [15] reported comparable perinatal outcomes between autologous and donor cycles after propensity score matching. Jiang et al. [13] noted that individualized endometrial preparation (GnRH agonist or antagonist protocols) did not fully offset the age-related decline in LBR. Notably, Xi et al. [19] demonstrated that NICS-based euploid selection improved clinical pregnancy and live birth rates in RPL/RIF patients, with no specific age subgroup analysis reported. Overall, while PGT-A increases implantation and reduces miscarriage rates compared to untested embryos, maternal age remains a significant modifier of live birth success in most but not all studies, indicating the need for individualized patient counseling.

Risk of Bias Assessment

Six of the eight studies demonstrated a low overall risk of bias across all seven domains, including confounding, selection of participants, classification of interventions, deviations from intended interventions, missing data, measurement of outcomes, and selection of the reported result [13-16,18,20]. These six studies [13-16,18,20] had low ratings in each domain, indicating robust methodological quality. In contrast, Kato et al. [17] were judged to have a serious overall risk of bias due to serious confounding, moderate concerns regarding participant selection and missing data, and moderate risk in the selection of the reported result. Xi et al. [19] received a moderate overall risk of bias rating, driven by moderate confounding, moderate missing data, and moderate concern for selective reporting. No study was excluded from the review based on bias; however, findings from Kato et al. [17] and Xi et al. [19] are interpreted with greater caution in the overall evidence synthesis (Table 3).

**Table 3:** Risk of Bias Assessment (ROBINS I)

First Author (Year)	Confounding	Selection of Participants	Classification of Interventions	Deviations from Interventions	Missing Data	Measurement of Outcomes	Selection of Reported Result	Overall Risk of Bias
Jiang et al., [13] (2025)	Low	Low	Low	Low	Low	Low	Low	Low
Chen et al., [14] (2025)	Low	Low	Low	Low	Low	Low	Low	Low
Cozzolino et al., [15] (2024)	Low	Low	Low	Low	Low	Low	Low	Low
Lawrenz et al., [16] (2024)	Low	Low	Low	Low	Low	Low	Low	Low
Kato et al., [17] (2023)	Serious	Moderate	Low	Low	Moderate	Low	Moderate	Serious
Pantou et al., [18] (2022)	Low	Low	Low	Low	Low	Low	Low	Low
Xi et al., [19] (2022)	Moderate	Low	Low	Low	Moderate	Low	Moderate	Moderate
Tan et al., [20] (2021)	Low	Low	Low	Low	Low	Low	Low	Low

Discussion****

The present systematic review synthesized evidence from eight retrospective cohort studies to evaluate the impact of euploid embryo transfer and maternal age on implantation and live birth outcomes in IVF-ET. The key findings indicate that while PGT-A with euploid embryo transfer significantly improves implantation rates and reduces miscarriage compared to untested embryos, maternal age continues to exert a variable effect on live birth rates across studies. Specifically, three studies reported a significant decline in LBR with advancing maternal age despite euploid transfer [13,18], whereas two other well-conducted studies found no independent age effect after adjusting for embryo quality and endometrial preparation [14,16]. These discrepant findings highlight the complexity of reproductive aging beyond aneuploidy alone and suggest that maternal age influences outcomes through multiple biological pathways, including endometrial receptivity, mitochondrial function, and epigenetic alterations, which are not corrected by PGT-A.

Our finding that maternal age remains a negative prognostic factor in some but not all studies aligns with the broader literature. For instance, a large retrospective study by Franasiak et al. [21] using trophectoderm biopsy and NGS demonstrated that euploidy rates decline linearly with age, but even among euploid embryos, implantation rates were significantly lower in women over 40 years compared to those under 35 (51.4% vs. 68.9%, p<0.001) [21]. Similarly, a multicenter European study by Metwelly et al. [22] reported that after a single euploid FET, live birth rates decreased from 58% in women <35 years to 42% in women ≥40 years, despite identical embryo quality [22]. These external data support the conclusions of Jiang et al. [13] and Pantou et al. [18] in our review, reinforcing that age-related endometrial or ooplasmic factors persist after aneuploidy screening.

Conversely, the absence of an age effect reported by Lawrenz et al. [16] and Chen et al. [14] is also supported by existing literature. A prospective cohort study by Abdala et al. [23] found no significant difference in ongoing pregnancy rates following euploid FET between women aged 35-37, 38-40, and 41-42 years (adjusted OR 1.0, 0.9, and 0.8, respectively, p=0.32) after controlling for endometrial preparation and embryo morphology [23]. These external comparisons suggest that the discrepant findings in our review may be explained by differences in study populations, endometrial preparation protocols, and the specific age cut-offs used.

One of the most clinically relevant observations from our review is the divergent utility of PGT-A between RPL and RIF patients. Kato et al. [17] demonstrated a dramatic benefit for RPL patients (LBR 80% vs. 0%, p=0.005) but no benefit for RIF patients. The biological rationale is that RPL is often driven by high rates of embryonic aneuploidy, whereas RIF is more frequently associated with endometrial factors, uterine anomalies, or immune dysregulation that are not addressed by embryonic ploidy status. Our review extends this by also including Xi et al. [19], who showed that NICS improved outcomes in RPL/RIF, although with a moderate risk of bias.

The role of blastocyst morphology as a predictor of live birth, independent of maternal age, was another important finding from Chen et al. [14] and Lawrenz et al. [16]. This aligns with a large retrospective analysis by Cimadomo et al. [24] of over 6,000 euploid FET cycles, which demonstrated that trophectoderm grade was significantly associated with LBR (OR 1.43 for good vs. fair, 95% CI 1.25-1.64), whereas maternal age showed no association after adjusting for ploidy and grade [24]. These external data strongly support the conclusion that embryo morphology remains clinically relevant even after PGT-A and that age alone should not be the sole determinant of transfer decisions.

Another key finding from our review is that oocyte origin (autologous vs. donor) in women with advanced maternal age did not significantly affect implantation, LBR, or miscarriage rates after single euploid FET, as reported by Cozzolino et al. [15]. This is also consistent with a propensity-score-matched study by Cimadomo et al. [24], which compared autologous and donor oocyte euploid FETs in women over 40 years and found no significant differences in LBR (45.2% vs. 47.6%, p=0.63) or miscarriage (14.5% vs. 13.9%, p=0.81) [24]. Another study by Park et al. [25] reported similar findings, concluding that once a euploid embryo is generated, the endometrium of an aged recipient does not inherently impair implantation compared to a younger donor oocyte recipient, provided that endometrial preparation is optimized [25]. This has important clinical implications: women with AMA considering donor oocytes can be reassured that, following euploid transfer, pregnancy outcomes are comparable to autologous cycles, and the decision should therefore focus on oocyte yield and patient preference rather than anticipated implantation failure. Future prospective multicenter studies with standardized age cut-offs, cumulative live birth reporting, and inclusion of endometrial receptivity biomarkers are needed to refine patient selection for PGT-A. Until then, clinicians should adopt an individualized approach, balancing the potential benefits of aneuploidy reduction against the risks of embryo discarding, false-positive results, and added financial burden.

The impact of endometrial preparation protocol on outcomes was noted by Lawrenz et al. [16] and Jiang et al. [13], who found that natural cycles, HRT, and individualized GnRH agonist/antagonist protocols did not fully offset age-related declines. More recently, a randomized controlled trial by Liu et al. [26] comparing natural cycle FET versus HRT FET in ovulatory women found equivalent live birth rates (46.7% vs. 44.9%, p=0.58), suggesting that protocol choice may be less critical than consistent optimization of endometrial thickness and timing [26]. However, none of the studies in our review specifically examined personalized embryo transfer (pET) guided by endometrial receptivity array (ERA), which is an important area for future research, particularly in older patients or those with RIF.

The use of NICS as an alternative to trophectoderm biopsy, as reported by Xi et al. [19], represents a promising but still investigational approach. Our external literature search identified a prospective cohort study by Chen et al. [27] comparing NICS versus TE biopsy in 350 cycles, which reported moderate concordance (78.5%) and similar ongoing pregnancy rates (51.2% vs. 52.8%, p=0.76), but with significantly lower cost and no risk of biopsy-related damage [27]. Therefore, while NICS may have a future role, especially in settings where biopsy is technically challenging or culturally unacceptable, the evidence base remains immature, and our finding of moderate risk of bias for Xi et al. [19] reflects these concerns.

Another important nuance from our review is the distinction between implantation rate, clinical pregnancy rate, and live birth rate. Several studies reported high implantation rates (up to 69.6% in RIF patients, Pantou et al. [18]) but lower live birth rates, particularly in AMA patients (as low as 35.3% in the same study). This discrepancy is largely explained by increased miscarriage rates in older women, even after euploid transfer. The biological mechanisms underlying euploid miscarriage in older patients include endometrial senescence, vascular insufficiency, altered immune tolerance, and epigenetic dysregulation of imprinted genes. This underscores that improving outcomes in AMA patients will require not only embryonic aneuploidy screening but also adjunctive therapies targeting endometrial receptivity, such as platelet-rich plasma, granulocyte colony-stimulating factor, or endometrial scratch.

The clinical implications of our review are several. First, for women over 38 years old undergoing IVF, PGT-A with a single euploid FET should be offered, but patients should be counseled that live birth rates will still be lower than in younger women, and miscarriage risk remains elevated. Second, for women with RPL, PGT-A is strongly supported by evidence from our review and external studies, particularly when balanced translocations or recurrent aneuploidy are suspected. Third, for RIF patients, PGT-A is not recommended as a first-line intervention; instead, evaluation of the endometrium (chronic endometritis, ERA, and microbiome) and uterine cavity should take priority. Fourth, embryo morphology remains prognostically important even after PGT-A, and, therefore, good-quality blastocysts should be prioritized for transfer. Fifth, autologous and donor oocyte cycles achieve comparable outcomes following euploid transfer in AMA patients, which may inform counseling for women considering oocyte donation.

Limitations

The present systematic review has several limitations that must be acknowledged. First, all eight included studies were retrospective cohort designs, which are inherently susceptible to selection bias, confounding by indication, and incomplete data reporting. Despite our use of the ROBINS‑I tool, which identified low risk of bias in six studies and moderate-to-serious risk in two, residual confounding cannot be entirely excluded. Second, there was substantial heterogeneity across studies in terms of maternal age cut-offs, PGT-A methodologies (NGS, aCGH, and NICS), endometrial preparation protocols, and outcome definitions (clinical pregnancy, ongoing pregnancy, and live birth), which precluded meta-analysis and limited the generalizability of pooled conclusions. Third, none of the studies reported on cumulative live birth rates per oocyte retrieval cycle, which is the most patient-relevant outcome in IVF; instead, all focused on per-transfer outcomes, potentially overestimating the benefit of PGT-A by excluding cycles that failed to produce any euploid embryos. Fourth, the follow-up duration for live birth and neonatal outcomes was variable and often incomplete, with only Cozzolino et al. [15] and Kato et al. [17] reporting birth weight or child development data. Fifth, publication bias may be present, as studies reporting positive or significant findings are more likely to be published, and all included studies were published in English-language journals, potentially excluding relevant non-English literature. Sixth, the sample sizes in subgroup analyses (e.g., RIF patients in Kato et al. [17], donor cycles in Cozzolino et al. [15]) were relatively small, leading to wide confidence intervals and imprecise effect estimates. Seventh, no study in our review performed a formal sample size calculation for subgroup comparisons, raising the possibility of type II errors (false negatives) for some null findings. Finally, our review did not include cost-effectiveness analyses, which are critical for health policy decisions regarding PGT-A implementation, nor did it address the psychological impact of extended embryo culture, biopsy, and genetic testing on patients.

## Conclusions

This systematic review of eight retrospective cohort studies demonstrates that euploid embryo transfer significantly improves implantation and reduces miscarriage rates compared to untested embryos, but maternal age continues to influence live birth outcomes in a subset of patients, particularly those aged 38 years and older. The benefit of PGT-A varies substantially by clinical indication: it is strongly supported for recurrent pregnancy loss, equivocal for advanced maternal age, and not supported for recurrent implantation failure. Blastocyst morphology and endometrial preparation remain important modifiable factors independent of ploidy status. Oocyte origin (autologous vs. donor) does not affect outcomes after euploid transfer in advanced maternal age patients.
